# SARS-CoV-2 spike protein induces a differential monocyte activation that may contribute to age bias in COVID-19 severity

**DOI:** 10.1038/s41598-022-25259-2

**Published:** 2022-12-02

**Authors:** Ines Ait-Belkacem, Celia Cartagena García, Ewa Millet-Wallisky, Nicolas Izquierdo, Marie Loosveld, Isabelle Arnoux, Pierre-Emmanuel Morange, Franck Galland, Nathalie Lambert, Fabrice Malergue, Jean-Marc Busnel

**Affiliations:** 1 Department of Research and Development, Beckman Coulter Life Sciences-Immunotech, Marseille, France; 2 Aix Marseille Université CNRS INSERM CIML Centre d’Immunologie de Marseille-Luminy, Marseille, France; 3 INSERM UMRs 1097, Aix Marseille University, Marseille, France; 4 Hematology Laboratory, Timone University Hospital, APHM, Marseille, France

**Keywords:** Cell biology, Immunology, Biomarkers, Diseases, Risk factors

## Abstract

A strong bias related to age is observed in COVID-19 patients with pediatric subjects developing a milder disease than adults. We hypothesized that a specific SARS-CoV-2 effect conjugated with preexisting differences in the immune systems may explain this. Using flow cytometry, we investigated basal immune differences in a cohort consisting of 16 non-infected young and 16 aged individuals and further leveraged an in vitro whole blood model of SARS-CoV-2 infection so that functional differences could be mined as well. In short, blood diluted in culture media was incubated 5 or 24 h with the trimeric spike protein or controls. Following unsupervised analysis, we first confirmed that the immune lymphoid and myeloid systems in adults are less efficient and prone to develop higher inflammation than those in children. We notably identified in adults a higher CD43 lymphocyte expression, known for its potentially inhibitory role. The spike protein induced different responses between adults and children, notably a higher increase of inflammatory markers together with lower monocyte and B cell activation in adults. Interestingly, CD169, a CD43 ligand overexpressed in COVID-19 patients, was confirmed to be strongly modulated by the spike protein. In conclusion, the spike protein exacerbated the preexisting lower immune responsiveness and higher inflammatory potential in adults. Altogether, some of the markers identified may explain the marked age bias and be predictive of severity.

## Introduction

COVID-19 disease is a global health threat that remains poorly understood. It is often associated with asymptomatic and mild symptoms but may progress to severe pneumonia leading to ARDS (acute respiratory distress syndrome). These severe cases require intensive care unit (ICU) admission and/or respiratory assistance^1^.

This disease is caused by the severe acute respiratory syndrome coronavirus (SARS-CoV-2), which is composed of a single-stranded RNA and a nucleocapsid (N) protein. Both are encapsulated in an envelope that contains different proteins including the spike protein (S)^2^.

When entering into the upper respiratory tract of the lung, the spike protein encounters and binds to the angiotensin-converting enzyme 2 receptor (ACE2). Largely expressed on the lung epithelium, ACE2 allows the virus to enter host cells^3^. SARS-CoV-2 infection leads to major releases of pathogen associated molecular patterns (PAMPs) and damage associated molecular patterns (DAMPs) that activate innate immune cells through their PRRs (pathogen-recognition receptors). Thus, endosomal toll-like receptor 7 (TLR7), TLR3, and cytosolic RIG-like receptor (RLR) can detect single-stranded RNA (ssRNA) and induce the activation of interferon regulatory factor 3 (IRF3), IRF7 and Nuclear factor kB (NF-kB). This leads to the rapid production of type I and III interferons (IFN), and pro-inflammatory cytokines^4^. Finally, the subsequent expression of interferon stimulated genes (ISGs) leads to key antiviral activity and effective innate and adaptative immune responses. The direct detection of IFN is challenging because of its low concentration and rapid clearance. Thus, measuring ISGs such as CD169 by flow cytometry may conveniently mirror IFN levels^5,6^. The sialoadhesin CD169 (Siglec-1) is a cell adhesion molecule found on the surface of macrophages and IFN-I activated monocytes^7^.

Some reports have shown that IFN response is blunted in severe forms of COVID-19 either through cellular mechanisms^8^, IFN deficiencies^9^, or auto-IFN-I antibodies^10^. In contrast, we and others have observed a very high expression of the ISG CD169 in almost all COVID-19 patients^11–16^. This suggests that other pathways may be implicated in CD169 upregulation. Previous reports showed that SARS-CoV-2 can induce the direct activation of monocytes and macrophages through spike interaction with TLR4 and TLR2 on cell lines and mouse models^17,18^. Recently, Minutolo and colleagues observed CD169 upregulation upon in vitro stimulation of PBMCs with SARS-CoV-2 spike protein^14^.

Others have shown SARS-CoV-2-infection to be associated with abnormal immune response. Most severe phenotypes have one or more of three characteristics. First, they may be characterized by lung tissue damage. Second, leucocyte subpopulation dysregulation is common (lymphopenia, basopenia, eosinopenia and/or increased neutrophil count). Third, there is often an over production of pro-inflammatory cytokines and infection/inflammation-related biomarkers such as C-reactive protein (CRP)^19–21^.

Comorbidities like diabetes, hypertension, and obesity are overrepresented in severe COVID-19 forms. Still, a strong and independent age bias is observed with pediatric patients developing milder diseases than adults^22–24^. It is known that immune system exhaustion and inflammatory potential increases with age. This phenomenon is also called inflamm-aging and could explain the severity bias^25^. However, despite the cross-disciplinary progress achieved over the last months, to the best of our knowledge, it remains unclear by which mechanism SARS-CoV-2 virus specifically induces much more severe forms in adults, in comparison with other viruses. In an attempt to address this question, we focused on previously described parameters associated with COVID-19 disease severity and especially on monocytes since there are increasing evidences showing that they may orchestrate dysregulated immune responses^26,27^. We performed an exhaustive flow cytometry immunophenotyping in two non-infected cohorts of adults and children to identify the best discriminant markers. In parallel, since spike protein has been shown to activate monocytes and macrophages, we developed an in vitro whole blood functional assay of SARS-CoV-2 infection. This model aimed at characterizing the immune responses to spike stimulation in the two groups, and further identifying potential segregating features.

## Material and methods

### Samples

The study was conducted in accordance with the Declaration of Helsinki and the French law on research involving humans. Residual ethylenediaminetetraacetic acid whole (EDTA) blood samples from 16 children (3–17 years old) and 16 adults (20–98 years old) were provided by the Biological Resources Center (CRB) of La Timone Hospital in May 2021. The study was approved by the ethical committee of the Assistance Publique—Hôpitaux de Marseille (AP-HM, certified NF S96-900 and ISO 9001 v2015) and by The French ministry of Health (authorization AC-2018-3105). Informed consents were obtained from the subjects aged above 18 years and from the parents/legally authorized representative of the subjects aged under 18 years. All samples were pseudonymized and subject care was not modified. Donors were not screened for anti-SARS-CoV-2 IgG or IgM levels to assess natural or vaccinal immunization status, but less than 10% individuals had been infected at this time in France^28^. Furthermore, samples were tested double negative for CD169 viral and CD64 bacterial markers and thus patients were considered as infection-free^29^.

### Extracellular staining

For immunophenotyping, an infection related panel together with panels dedicated to granulocytes, T cells, B cells and dendritic cells (Table 1) were processed according to the recently developed rapid one-step method^29^. Briefly, whole blood was simultaneously lysed and stained in a lysing/fixative buffer (Versalyse containing Fixative solution 0,05X, Beckman Coulter, Brea, USA) for at least 20 min.

**Table 1:** Flow cytometry panels used in the study.

	Panel	Commercial/ prototype	Conjugates	Format	Cytometer
Phenotyping	Infection related panel (surface)	Myeloid activation test + CD43	CD169-PE, HLA-DR-APC, CD64-PB, and CD43-AF750	Liquid	NAVIOS
	Granulocyte panel (surface)	DURAClone IM Granulocytes	CD294-FITC, CD16-ECD, CD33-PC5.5, CD11b-PC7, CD274-APC, CD3-CD19-CD56-CD14 APC-AF700, CD62L-APC-AF750, CD15-PB, and CD45-KrO	Dried	NAVIOS
	T cell subset panel (surface)	DURAClone IM T cell subset	CD45RA-FITC, CD197-PE, CD28-ECD, CD279-PC5.5, CD27-PC7, CD4-APC, CD8-AF700, CD3-APC-AF750, CD57-PB, and CD45-KrO	Dried	NAVIOS
	B cell subset panel (surface)	DURAClone IM B cells	IgD-FITC, CD21-PE, CD19-ECD, CD27-PC7, CD24-APC, CD38-APC-AF750, IgM-PB, and CD45-KrO	Dried	NAVIOS
	Dendritic cell panel (surface)	DURAClone IM Dendritic cells	CD16-FITC, CD3-CD14-CD19-CD20- CD56-PE, CD1c-PC5.5, CD11c-PC7, Clec 9A-APC, CD123-APC-AF700, HLA-DR-PB, and CD45-KrO;	Dried	NAVIOS
	Treg panel (intracellular)	DURAClone IM Treg	CD45RA-FITC, CD25-PE, CD39-PC5.5, CD4-PC7, FoxP3-A647, CD3-APC-AF750, Helios-PB, and CD45-KrO	Dried	NAVIOS
Phenotyping + functional analysis	Leukocyte activation panel (surface)	Prototype	CD54-FITC, CD11b-PE, CD16-ECD, CD56-PC5.5, CD69-PC7, CD62L-APC, tmTNF-AF700, CD3-CD66b-APC-AF750, CD14-PB, and CD45-KrO	Liquid	CytoFLEX
	Granulocyte activation panel (surface)	Prototype	DHR123-FITC**, MPO-PE, CD62L-ECD, CD11b-PC7, CD294-AF647, CD16-APC-AF700, CD66b-APC-AF750, DAPI-PB, and CD45-KrO	Liquid	CytoFLEX
	Cytokine activation panel (intracellular)	Prototype	IL8-A488*, IL6-PE*, CD14-ECD, IL4-PC7, CD3-APC, TNFa-A700, IFNγ-PB, and CD8-CD45-KrO	Liquid	NAVIOS
	Infection related panel (surface)	Myeloid activation test + CD43	CD169-PE, HLA-DR-APC, CD64-PB, and CD43-AF750	Liquid	NAVIOS

All the products are from Beckman Coulter except those indicated: *Product from Biolegend (San Diego, USA), **Product from Invitrogen (Waltham, USA).

Systemic activation panel was processed as follow: after 30 min of staining, OptiLyseC was added to lyse red blood cells following the manufacturer's instruction (Beckman Coulter, Brea, USA).

Granulocyte activation panel was treated as follow: after 30 min of staining, VersaLyse was added to lyse red blood cells following the manufacturer's instruction (Beckman Coulter, Brea, USA).

For all panels, cells were concentrated by centrifugation for 5 min at 150*g* and resuspended in Phosphate-Buffered Saline (PBS) before flow cytometry analysis.

### Intracellular staining

Cytokine and regulatory T cell (Treg) panels were processed by fixing and further permeabilizing the cells following Perfix-nc manufacturer's instructions for use (Beckman Coulter, Brea, USA). All samples were concentrated by centrifugation (300*g*, 5 min) and resuspended in PBS prior to analysis.

### Cell culture and functional assay

EDTA blood activation was possible after a 12.5-fold blood dilution in RPMI 1640 medium (Sigma-Aldrich, St. Louis, USA) containing 10% decomplemented Fetal Calf Serum (FCS, Sigma-Aldrich, St. Louis, USA), 1 mM of a calcium chloride solution (CaCl_2_, Sigma-Aldrich, St. Louis, USA) and heparin at a concentration of 1.5 × 10^–3^ mg/mL (Sigma-Aldrich, St. Louis, USA). Besides enabling EDTA blood to be activated, the blood dilution step reduced the potential contribution of circulating SARS-CoV-2 antibodies that might have interfered with cell activation. Calcium pre-treatment countered EDTA-calcium chelation that prevents cell polarization and activation. Brefeldin A (5 μg/mL, Sigma-Aldrich, St. Louis, USA) was added for intracellular staining. Samples were treated with lipopolysaccharide (LPS, 2 mg/mL, Sigma-Aldrich, St. Louis, USA), saturating dose of SARS-CoV-2 recombinant trimeric spike protein (5 nM, Acro Biosystems, Newark, USA), or PBS as a negative control. Samples were incubated at 37 °C in a 5% CO_2_ incubator for 5 or 24 h (only for the infection-related markers study). Cells underwent surface and intracellular staining as previously described using the following panels: leukocyte activation, granulocyte activation, cytokine, and infection-related panels.

### Flow cytometry and statistical analysis

Samples were acquired either on a 10-color, 3-laser NAVIOS or a 13-color 3-laser CytoFLEX cytometer, both from Beckman Coulter.

Data treatment was performed with Kaluza Analysis 2.1 software (Beckman Coulter, Brea, USA) and statistical analyses were generated with JMP 14.2.0 (SAS Institute, Cary, USA). Marker mean fluorescence intensities (MFI) or subset percentages were studied. Unsupervised analysis of considered parameters was performed using the response screening platform that yielded most discriminative features based on ANOVA p-values and false discovery rate (FDR) corrected p-values. Principal component analysis (PCA) and hierarchical clustering were also conducted. Box plot representations and nonparametric Wilcoxon Rank Sum tests, equivalent to Mann–Whitney tests, were also performed to compare parameter levels across different subgroups of individuals. Statistical significances were established according to p-values lower than or equal to 0.05 (*). All p-values higher than 0.05 were considered statistically non-significant (ns).

## Results

### Immunophenotyping

**Figure 1:**
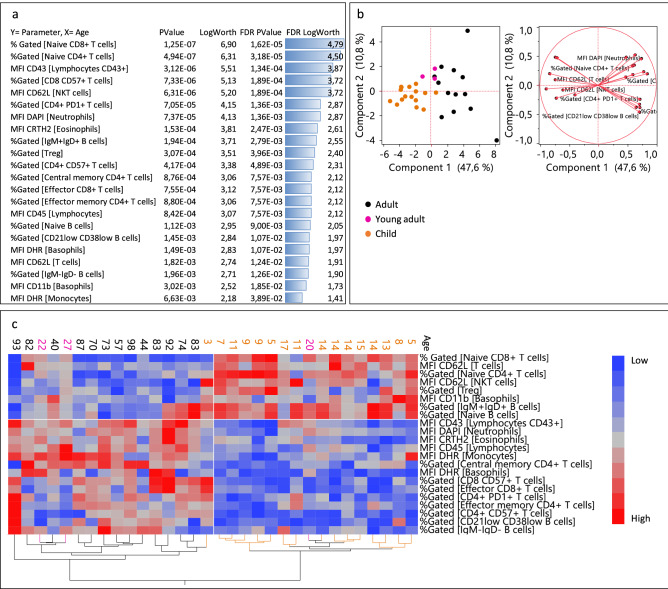
Unsupervised analysis of the biological parameters considered: (**a**) response screen of the features as the function of the age showing the most discriminant factors. (**b**) PCA analysis of the best discriminators. (**c**) Hierarchical clustering of the best factors. Orange dots represent children, black adults, and pink young adults. Parameters in blue are downregulated and parameters in red are upregulated.

It is known that basal immune characteristics are different between adults and children^25^. In order to identify the stronger discriminators between the two groups, eight flow cytometry panels (4–10 colors) were considered to phenotype 16 children and 16 adults (infection-free). While 129 parameters were considered in total, an unsupervised analysis yielded 22 discriminative features between the two age groups with FDR p-values equal or lower than 0.05 (Fig. 1a). Besides already described naïve CD4 and CD8 T cell subsets, lymphocyte CD43 expression was ranked as the third best discriminator between cohorts. The 22 identified discriminators were further used for PCA analysis, resulting in an effective separation of children and adults (Fig. 1b). Together with a higher heterogeneity among adults, we observed that within this group, young adults (ranging from 20 to 30 years old) were located close to the children group. The children’s group was better clustered with only the youngest child (three years old) outside the group.

To further investigate whether these parameters were up or downregulated in adults, we leveraged a hierarchical clustering analysis that showed that the adult group was characterized by less abundant naïve (naïve CD8 + T, naïve CD4 + T, IgM + IgD + B cells, naïve B cells, CD62L + T, and NKT cells) and regulatory compartments (Treg) (Fig. 1c). Accordingly, adults presented larger memory and effector compartments (central memory CD4 + T cells, CD21low CD38low B cells, IgM- IgG- B cells, effector CD8 + T cells, and effector memory CD4 + T cells), more senescent T cells (CD8 + CD57 + T cells and CD4 + CD57 + T cells) and exhausted T cells (CD4 + PD1 T cells). CD43 and CD45, which are both involved in lymphocyte activation and regulation were found to be more expressed in adults than in children.

Beyond the lymphoid compartment, some differences, such as higher oxidative burst rates (DHR) and more apoptotic cells (DAPI +) in adults were also observed in the myeloid subsets. A lower CD11b expression on adult basophils was found, together with a higher eosinophil CD294 prostaglandin D2 receptor (CRTH2), both potentially related to the Th2 response. As illustrated in Supplementary Fig. S1, it was interesting to find significant correlations between a variety of immune parameters and the age of the individuals.

### Cell function

**Figure 2:**
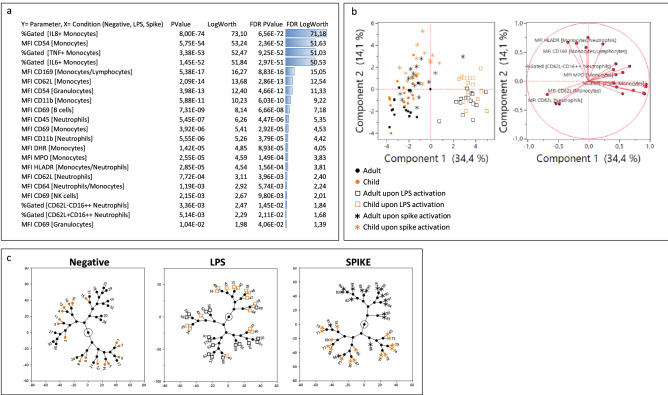
Unsupervised analysis of the biological parameters considered: (**a**) response screen comparing non-stimulated, LPS and spike conditions showing the most discriminant features. (**b**) PCA analysis of the best discriminators. (**c**) Constellation clustering analysis of the best factors. Orange color represents children and black adults. Squares illustrate LPS condition and asterisks spike condition.

Next, in an effort to capture hypothetic functional differences between the two age groups, whole blood from the same 16 children and 16 adults was activated with a recombinant trimeric SARS-CoV-2 spike protein. Four flow cytometry panels were considered for the functional characterization of the samples. As positive controls, LPS induced TNFα upregulation whereas IFN I induced CD169 expression. Nucleocapsid and non-trimeric spike had no effect and therefore constituted additional negative controls (Supplementary Fig. S2). LPS was chosen as a reference of monocyte activation for further analysis since spike protein interacts with TLR4 on monocytes^18^. Moreover, it has been shown that SARS-CoV-2 seems to mimic a bacterial infection through the induction of bacterial markers such as CD64^30^. Like previous results^14,18^, our model showed a strong monocyte response to trimeric spike activation, as demonstrated by TNFα production and CD169 upregulation. In total, 21 parameters out of the 83 analyzed were found to significantly vary across the three considered conditions (non-stimulated, LPS, or trimeric spike) (Fig. 2a). These parameters were selected for PCA analysis and not only provided prominent separation between the three conditions tested, but also enabled a substantial separation between the two age groups (Fig. 2b). On this latter point, the trimeric spike condition yielded the strongest discrimination between the two age groups, as exhibited by a hierarchical clustering experiment (Fig. 2c).

To further elucidate the differences between LPS and trimeric spike activation, two unsupervised analyses were performed comparing LPS and trimeric spike conditions to negative control. As expected, LPS response was characterized by high IL-8, IL-6 and TNFα monocyte production correlated with a high monocyte activation (CD69), oxidative burst (DHR, MPO) and dysregulation of other adhesion molecules (CD62L, CD11b, CD54). Granulocytes were also activated, through direct or indirect signaling, showing dysregulated CD54, CD45, CD11b, CD62L, CD64, CD16, MPO, DHR and DAPI.

**Figure 3:**
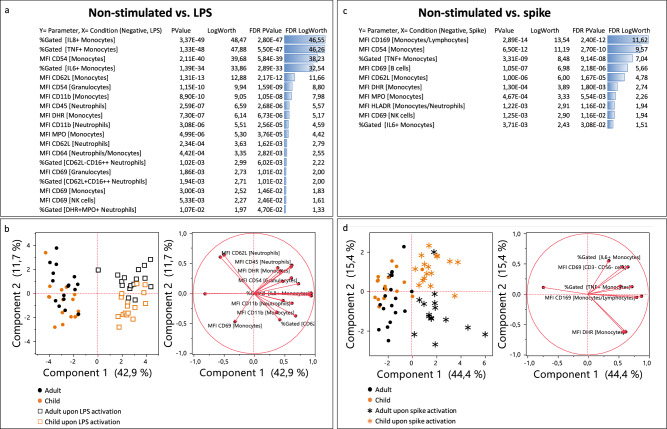
Unsupervised analysis of the biological parameters considered: (**a**) response screen comparing non-stimulated to LPS conditions (**a**) and non-stimulated to trimeric spike conditions (**c**) showing the most discriminant features. (**b**,**d**) PCA analysis of the best discriminators. Orange color represents children and black adults. Squares illustrate LPS condition and asterisks spike condition.

The trimeric spike protein induced a monocytic response characterized by TNFα and IL-6 production along with upregulation of CD54, DHR, and MPO and decreased CD62L. However, as suggested already by the eigenvectors of Fig. 2b, the trimeric spike response could be distinguished from the LPS one by strong monocyte CD169 and HLA-DR increases. A much weaker granulocyte activation (all parameters having FDR p-values higher than 0.05) could also be observed. Interestingly, a higher activation of B cell subset (CD69) also characterized the response to trimeric spike protein (Fig. 3 and Supplementary Fig. S3).

**Figure 4:**
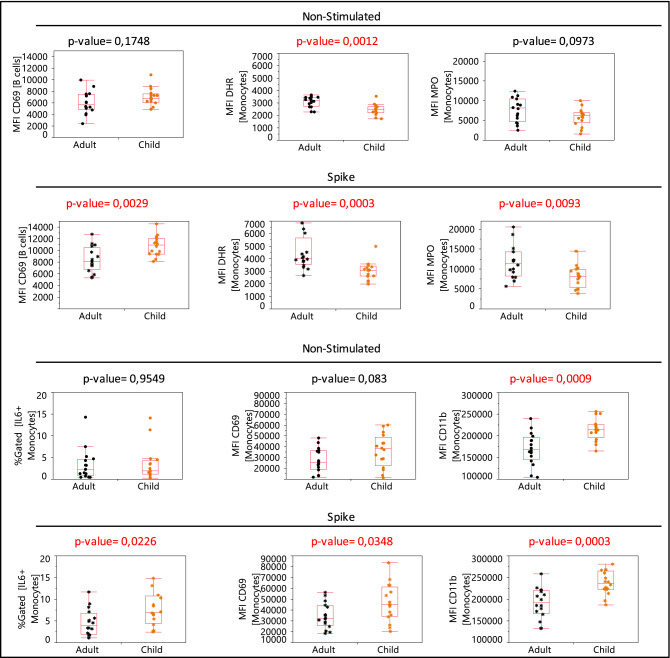
Representative box plots of the 6 parameters that differentially respond to spike stimulation (p-value equal or lower than 0.05) between adults and children. p-value in red color illustrates significative differences between child and adult group.

Since the trimeric spike condition provided a better separation between adults and children (Fig. 2c), we next focused on the trimeric spike condition to investigate possible functional differences existing between the two age groups. Box plot illustrations showed the 6 parameters responding to the trimeric spike activation (p-value equal or lower than 0,05) that were significantly different between adults and children: lower activation of B cells (CD69) was found in adults with lower monocyte response (IL6, CD69, and CD11b) and further increase of DHR and MPO on monocytes (Fig. 4).

## Discussion

A strong bias linked to age is observed in COVID-19 disease. As the immune system plays a key role in the progression of the disease, we hypothesized that a specific SARS-CoV-2 mode of action conjugated with preexisting differences in their immune systems may explain this bias. To study these differences, we performed exhaustive immunophenotyping and functional analysis in two non-infected cohorts of adults and children to identify markers segregating the two groups.

Multiple flow cytometry panels optimized for fresh whole blood investigation combined with easy processing methods enabled an unbiased and robust study of 129 phenotypic markers on all major leukocyte populations. Minor leukocyte populations such as innate lymphoid cells (ILC) were not deeply investigated in this study^31^. Also, plasmacytoid DC were specifically labeled using CD123, with a small risk of including some pre-DC, or AXL + SIGLEC6 + DC (AS-DC) which would have been better eliminated using more specific markers such as CD303 or CD304^32,33^.

We confirmed that adults are characterized by a reduced naïve and regulatory lymphoid compartment, a developed effector, memory, senescent, and exhausted lymphocyte set as well as a high oxidative burst rate, in agreement with literature that describes decreased immune function of lymphocytes upon aging^34^, and the inflamm-aging phenomenon^35^. Our results also corroborated with less widely described features such as an increased CD43 and CD45 expression on lymphocytes with age^36,37^. Both markers have been shown to be involved in T cell regulation, whose functions are controversial since they may activate or inhibit T cells^38–40^. Notably, we found that CD43 lymphocyte expression was one of the best discriminators. Interestingly, higher CD43 in adults has been associated with lower response efficiency to RSV infection, proposing that monocyte CD169 binding to the highly expressed CD43 reduces RSV-induced IFN-γ release by adult T cells^36^.

Moreover, lymphocytes from adults expressed less CD62L adhesion molecule suggesting that adults’ patrolling capabilities may be less efficient as CD62L is required for cell homing to lymph nodes^41^. Of note and potentially related to the COVID-19 pathogenicity, CD62L low lymphocytes tend to migrate preferentially to the lungs^42^. Overall, these results indicate that the adults’ immune lymphoid system is less efficient in comparison to children.

A deep myeloid compartment analysis showed higher apoptotic neutrophils with higher oxidative burst rate in adults. We found lower CD11b expression in adult basophils as compared to children, which may further explain the potential of adults to develop an uncontrolled inflammation as CD11b is proposed to play a critical role in limiting unwanted hyperinflammation via phagocytosis and anti-inflammatory cytokine production^43^. Interestingly, a previous report showed that a subset of neutrophils producing H2O2 is able to inhibit T cell responses through the dimer CD11b/CD18 (Mac-1)^44^. These results agree with the exhausted immune system in adults and reinforces its link with the severity of COVID-19.

We also observed lower basal eosinophil CRTH2 expression in the children group. As a receptor for PGD2, we hypothesized that CRTH2 could be perceived as a susceptibility factor for inflammatory events with higher levels correlating with a higher risk for more dramatic events. Previous studies have shown that CRTH2 expression on both basophils and eosinophils was significantly lower in severe forms of COVID-19, suggesting that CRTH2 downregulation may occur during an extreme inflammation event^45^. Altogether, these results indicate that adult’s immune myeloid system is prone to develop higher inflammation environment compared to children.

Also, not only considering differences between children and adults but also heterogeneity among individuals, some of the markers identified could be predictive of more severe cases. As an example, CD43 is globally higher in adults than children, with children having low or medium CD43 levels and adults having medium or, in 4 out of 16 cases, high CD43 levels. (Supplementary Fig. S1) Testing this hypothesis deserves a study on a larger prospective cohort.

We wondered how SARS-CoV-2 virus may interact with these two different preexisting immune systems. It is unclear whether the ACE2 receptor is expressed on leucocytes^46,47^. However, it has been shown that SARS-CoV-2 can induce monocyte/macrophage activation through direct trimeric spike interaction with TLR4 and TLR2. This interaction induces significant IL-1β and IL-6 production through Myd88 and NFkB signaling pathways^17,18^. Other signaling pathways might be triggered upon monocyte activation in accordance with recent studies in which human monocytes from PBMCs were activated with the trimeric spike protein inducing IL6 and IL 10 release, and surprisingly both ISG CD169 (Siglec-1) and HLA-DR^14^. Still, these observations were limited to PBMCs, cell lines or mouse models. In our study, we reproduced this monocyte activation using a whole blood model, finding IL6, but also TNFα production. This supports TLR4 and/or TLR2 signaling.

Our data further confirmed CD169 and HLA-DR upregulation upon trimeric Spike activation. CD169 is an ISG that has been recently recognized as very convenient for IFN I monitoring^5^. In addition, CD169 expression induced by IFN I production is an early marker of acute viral infection, and more importantly, of SARS-CoV-2 infection since it is more expressed in COVID-19 disease than in other respiratory viral infections^12,48^. It is tempting to speculate that CD169 overexpression in COVID-19 patients may occur as a result of a combined IFN I response and SARS-CoV-2 spike protein contact with monocytes. Interestingly, van den Berg and colleagues showed that CD169 interacts with CD43^49^. We and others have observed a higher lymphocyte CD43 expression in adult than in children^36^. Despite controversial literature, CD43 engagement can potentially result in an inhibitory effect on lymphocytes^50^. These observations suggest that the high levels of CD43 in adults and CD169 characteristic of SARS-CoV-2 infection may trigger a higher lymphocyte inhibition resulting in lower capabilities to fight the virus in adults compared to children. This could be related to the lymphopenia typically observed in COVID-19 patients. We believe that this new theory should be further investigated since the CD169/CD43 interaction might be an interesting therapeutic target. Furthermore, CD169 (sialoadhesin) interaction with SARS-CoV-2 and other viruses such as HIV through their sialylated surface protein is known to mediate trans-infection what could further exacerbates the cell-to-cell viral transmission^51^.

Of note, most hospitalized COVID-19 patients are treated with corticosteroids, which are expected to reduce inflammation and avoid a cytokine storm. Interestingly, CD169 is decreased by corticoid in lupus patients and could play a role in this putative CD43/CD169 lymphocyte inhibition^52^.

Regarding TLR2 and/or TLR4 engagement with the trimeric spike protein, we and others have demonstrated monocyte activation through inflammatory cytokine release (IL-1β, IL-6 and TNFα)^17,18^. Interestingly, this interaction requires a trimeric form of the spike protein suggesting a crosslinking of receptors. In this study we observed modulation of activation markers on monocytes (CD69, CD54, MPO, DHR, and CD62L). Together with the ISG CD169, this observation points to another signaling pathway. It is known that the viral ligand/ TLR2 complex is internalized and activates IRF7/3 or IRF2/IRF1/STAT1 to upregulate IFN-β or IFN-α respectively^53^.

It is known that TLR4 mediates anti-bacterial immune responses by recognizing LPS from bacteria playing an important role in the development of sepsis^54,55^. In this bacterial infection context, it has been shown that CD64 increases on neutrophils in most bacterial infections^56^. Interestingly, this bacterial-related marker is increased upon SARS-CoV-2 infection confirming a dysregulated hyperinflammatory immune response^30^. In our study, we observed a trend towards a higher expression of CD64 in neutrophils upon trimeric spike activation although no significant differences were found. Altogether, we hypothesize that the SARS-CoV-2 virus may mimic a bacterial infection via TLR4 and further CD64 dysregulation. This would support the uncontrolled and inappropriate immune response characteristic of this condition.

Regarding the difference between adults and children upon trimeric spike activation, we have found a lower monocyte response (IL6, CD69, and CD11b), a higher increase of DHR and MPO on monocytes, and a lower activation of B cells (CD69) in adults. These findings agree with the exhausted and hyper-inflammatory immune system in adults.

This study has some limitations. First, it included a limited number of samples, thus it should be confirmed on a larger cohort. In addition, circulating IgG and vaccinal status may be recorded, ideally the type of IgG (infectivity enhancing or neutralizing) should even be taken in account as their implication in the anti-infectious response remains unclear^57^.

While we have compared non-trimeric and trimeric alpha variant of the SARS-CoV-2 spike protein, it would be interesting to consider in the future additional molecules. Spike from other coronaviruses such as SARS-CoV-1 or Middle East respiratory syndrome–related coronavirus (MERS), or even other SARS-CoV-2 variants for example represent very valuable perspectives to explore as the related mutations may be associated with disease severity^58^.

To conclude, our data suggest that a direct interaction of the viral spike protein with monocytes/macrophages via toll-like receptors could play a central role in SARS-CoV-2 pathogenicity. This interaction may induce and exacerbate different immune and inflammatory responses between children and adults who present pre-existing differences including ineffective and senescent lymphocytes, impaired regulation capabilities, as well as high oxidative and inflammatory granulocytes in adults. This may account for the marked age bias in disease severity, and we propose that some of the markers identified here could be predictive of COVID-19 severe forms. At least, our work demonstrate that whole blood assay represents a valuable approach to gather insights on pathological mechanisms.

## Supplementary Information

Supplementary Information.

## Data Availability

The authors confirm that the data supporting the findings of this study are available within the article and its supplementary materials.
