# Supplementary material for: SARS-CoV-2 spike protein induces a differential monocyte activation that may contribute to age bias in COVID-19 severity
Source: Sci Rep. 2022 Dec 2;12:20824. doi: 10.1038/s41598-022-25259-2 (PMC9716544; doi:10.1038/s41598-022-25259-2)
Supplement: Supplementary file 1 — Supplementary Information. [file 41598_2022_25259_MOESM1_ESM.docx]

**Supplementary figure 1:** Bivariate correlation analysis of the best discriminators with age. Orange dots represent children, black adults, and pink young adults.

**Supplementary figure 2:** TNFα and CD169 expression upon different stimulation conditions: Non-stimulated, LPS, INF I (β), nucleocapsid, non-trimeric spike, and trimeric spike, in all donors (a) and representative donors (b).

**Supplementary figure 3:** Representative box plots of the 10 best parameters responding to Spike stimulation (FDR p-value equal or lower than 0,05). Orange color represents children and black adults.
